# Moderate intensity supine exercise causes decreased cardiac volumes and increased outer volume variations - a cardiac magnetic resonance imaging study

**DOI:** 10.1186/1532-429X-16-S1-P38

**Published:** 2014-01-16

**Authors:** Katarina Steding-Ehrenborg, Robert Jablonowski, Per M Arvidsson, Marcus Carlsson, Håkan Arheden

**Affiliations:** 1 Cardiac MR Group Lund, Lund University, Skane University Hospital Lund, Lund, Sweden; 2 Copenhagen Muscle Research Centre, Rigshospitalet, Copenhagen, Denmark; 3 Danish Research Centre for Magnetic Resonance, Hvidovre Hospital, Copenhagen, Denmark

## Background

The effects on left and right ventricular (LV, RV) volumes during physical exercise remains controversial. Furthermore, no previous study has investigated the effects of exercise on longitudinal contribution to stroke volume (SV) and the outer volume variation of the heart. The aim of this study was to determine if LV, RV and total heart volumes (THV) as well as cardiac pumping mechanisms change during physical exercise compared to rest using cardiac magnetic resonance imaging (CMR).

## Methods

26 healthy volunteers (6 women) underwent cine CMR at rest and exercise. Exercise was performed using a custom built ergometer for one-legged exercise in the supine position during breath hold imaging. Cardiac volumes and atrio-ventricular plane displacement were determined. Heart rate (HR) was obtained from ECG.

## Results

**Figure 1:**
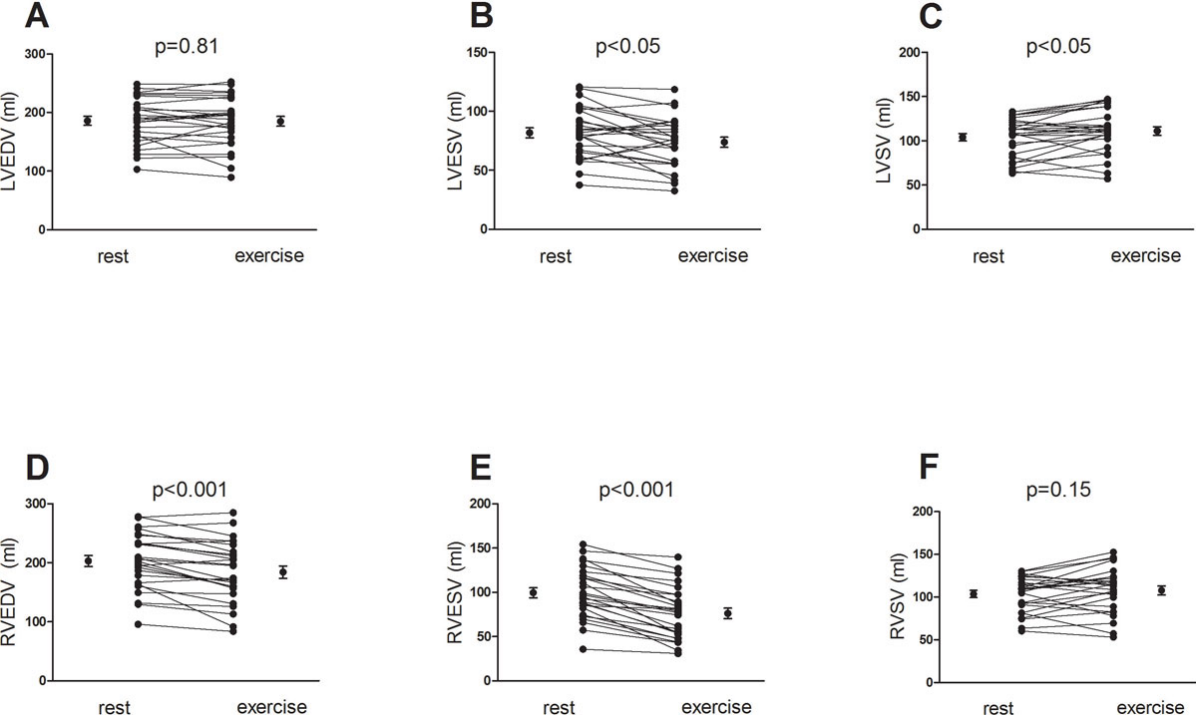
**Left and right ventricular volumes and stroke volumes at rest and exercise**. Upper panel shows no changes in left ventricular end-diastolic volumes (A) and a small but significant decrease in end-systolic volume (B), leading to an increased stroke volume (C). Lower panel show a significant decrease in right ventricular end-diastolic volume (D) and end-systolic volume (E). Right ventricular stroke volume increased during exercise, however not statistically significant (F). Error bars denote mean and standard error of the mean (SEM).

**Figure 2:**
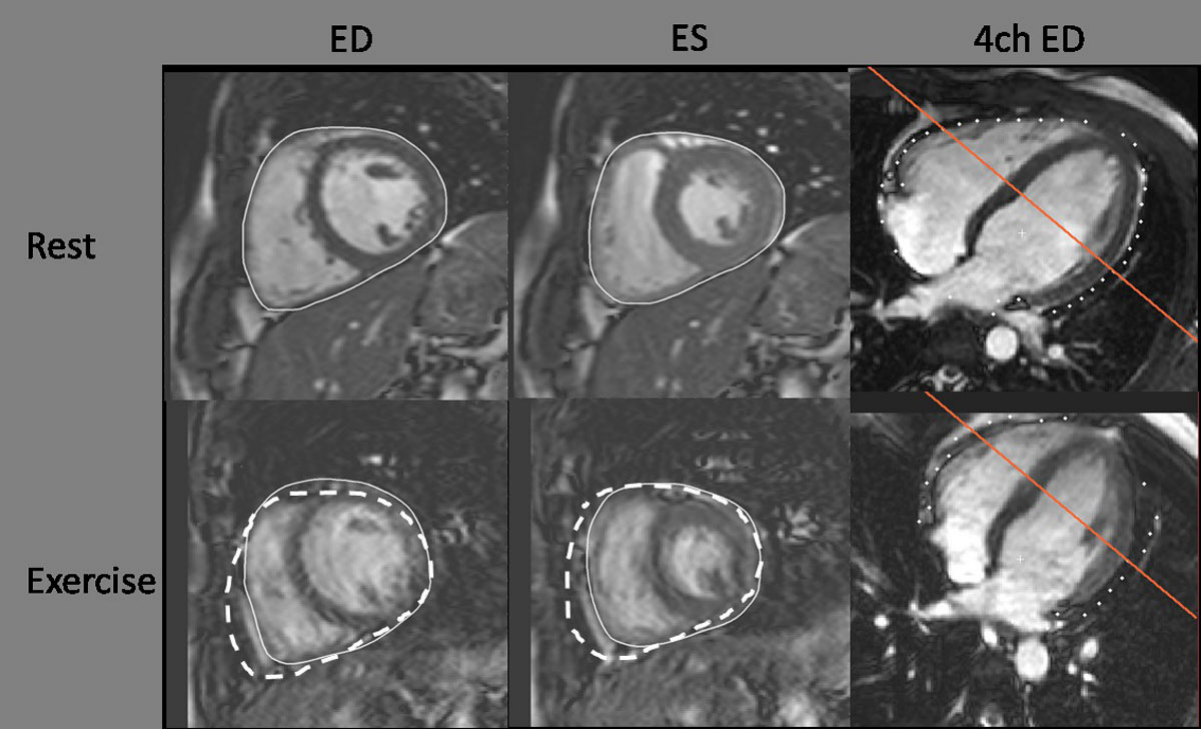
**Mid-ventricular short axis slices in end-diastole (ED) and end-systole (ES) during rest and exercise with the corresponding 4-chamber view (4 ch) to illustrate the location of the slice**. The solid line indicates delineations for total heart volume. In the exercise images, the dashed line shows the total heart volume delineation copied from the corresponding resting image. The right ventricular volume is decreased whereas the left ventricle remains unchanged.

HR increased during exercise (60 ± 2 to 94 ± 2 bpm, p < 0.001). LVEDV remained unchanged (p = 0.81) and LVESV decreased with -9 ± 18% (p < 0.05) causing LVSV to increase with 8 ± 3% (p < 0.05) (Figure 1A-C). RVEDV and RVESV decreased (-7 ± 10% and -24 ± 14% respectively, p < 0.001) and RVSV was increased with 5 ± 17% although not statistically significant (p = 0.18) (Figure 1 D-F). Longitudinal contribution to RVSV decreased during exercise (-6 ± 15%, p < 0.05) but was unchanged for LVSV (p = 0.74). THV decreased during exercise (-4 ± 1%, p < 0.01) and total heart volume variation (THVV) increased during exercise from 5.9 ± 0.5% to 9.7 ± 0.6%, p < 0.001) (Figure 2).

## Conclusions

Cardiac volumes and function are significantly altered during supine physical exercise. THV becomes significantly smaller due to decreases in RVEDV whilst LVEDV remains unchanged. THVV and consequently radial pumping increases during exercise which may improve diastolic suction during the rapid filling phase.

## Funding

This study was funded by the Swedish Research Council, the Swedish Heart and Lung Foundation, Region of Scania, the Medical Faculty at Lund University, Sweden, the Swedish Heart Association and Novo Nordisk Foundation, Denmark.

